# Correction to “Immunohistochemical localization of d‐*β*‐aspartic acid in congenital and acquired middle ear cholesteatoma”

**DOI:** 10.1002/lio2.1052

**Published:** 2023-04-13

Kitaya S , Ikeda R , Suzuki J , et al. Immunohistochemical localization of d‐*β*‐aspartic acid in congenital and acquired middle ear cholesteatoma. Laryngoscope Investigative Otolaryngology. 2022;7(4):1155–1163. doi: 10.1002/lio2.856 36000040PMC9392411

In the Abstract (Objective/Hypothesis), the second line needs to be revised from “We assessed the immunoreactivity to k‐*β*‐aspartic acid of congenital and acquired middle ear cholesteatomas.”

To “We assessed the immunoreactivity to d‐β‐aspartic acid of congenital and acquired middle ear cholesteatomas.”

We apologize for this error.

